# Immunocompetent C57BL/6 syngeneic mouse ovarian cancer models with defined genetic alterations

**DOI:** 10.1038/s41598-025-18960-5

**Published:** 2025-10-07

**Authors:** Hasmik Agadjanian, Beth Y. Karlan, Christine S. Walsh, Sandra Orsulic

**Affiliations:** 1 Women’s Cancer Program, Cedars-Sinai Medical Center, Los Angeles, CA USA; 2 Department of Obstetrics and Gynecology, David Geffen School of Medicine, University of California Los Angeles, Los Angeles, CA USA; 3 Jonsson Comprehensive Cancer Center, University of California Los Angeles, Los Angeles, CA USA; 4 Division of Gynecologic Oncology, Department of Obstetrics and Gynecology, University of Colorado, Aurora, CO USA; 5 Department of Pathology and Laboratory Medicine, David Geffen School of Medicine, University of California Los Angeles, Los Angeles, CA USA; 6 Department of Veterans Affairs, Greater Los Angeles Healthcare System, Los Angeles, CA USA

**Keywords:** Ovarian cancer, Mouse models of cancer, Syngeneic cancer cell lines, Cyclin E, p53, Immunogenic cell death, Cancer, Oncology

## Abstract

Ovarian cancer remains one of the deadliest gynecologic cancers globally, with limited progress in early detection and treatment. Syngeneic mouse ovarian cancer cell lines, derived from immunocompetent mice, have become essential tools for studying ovarian cancer biology and assessing novel therapies. With the rise of immunotherapies such as immune checkpoint inhibitors and cancer vaccines, these syngeneic models are critical for preclinical studies within the context of an intact immune system. The availability of diverse syngeneic ovarian cancer models ensures that research captures the full spectrum of human ovarian cancer variability, including variations in genetic mutations, signaling pathways, tumor antigenicity, and molecular subtypes. Here, we report the development and characterization of a panel of syngeneic ovarian cancer cell lines with defined combinations of initiating genetic alterations, such as TP53 deficiency, Hras mutation, and overexpression of Myc and Cyclin E. The Introduction of one or two oncogene drivers resulted in TP53-/- cell transformation and growth in nude and immunocompetent syngeneic C57BL/6 mice. Intraperitoneal tumors grew with high penetrance and had a wide metastatic distribution and concurrent ascites, which closely resembles the clinical picture of human serous ovarian cancer.

**Supplementary Information:**

The online version contains supplementary material available at 10.1038/s41598-025-18960-5.

## Introduction

Ovarian cancer is a heterogeneous disease characterized by late-stage diagnosis and poor survival rates. Research efforts are hindered by the complexity of the tumor microenvironment and the lack of suitable preclinical models. While immortal human cell lines and patient-derived xenograft (PDX) models have contributed significantly to understanding tumor biology, they are limited by their immunodeficient nature, which precludes the study of immune-mediated mechanisms. Genetically modified mouse models of ovarian cancer address this limitation^1^ but the long and unpredictable tumor latency is impractical for testing therapies. Syngeneic mouse models, using cell lines derived from the same genetic background as the host, provide a cost-effective immunocompetent platform for ovarian cancer research and drug testing^2^.

Several syngeneic ovarian cancer models have been established from spontaneous or chemically induced tumors in genetically inbred mice. Examples include ID8^3^, derived from C57BL/6 mice, and STOSE^4^, developed from BALB/c mice. These models exhibit varying degrees of aggressiveness and mimic distinct ovarian cancer subtypes. The ID8 cell line is the most commonly used syngeneic model in research and drug testing. Its ability to form tumors and ascites in mice makes it a practical model for evaluating treatment efficacy in conditions resembling high grade advanced human ovarian cancer. Despite its widespread use, there are several limitations to the ID8 cell line, which can impact the reliability and translational relevance of research findings. The ID8 model lacks key molecular features that drive high-grade serous ovarian cancer progression, such as TP53 mutations and BRCA-related DNA damage repair mechanisms. To overcome these limitations, researchers introduced Trp53, Brca1/2 Pten, and Nf1 mutations into ID8 cells^5,6^. Despite these improvements, the original ID8 cell line model continues to be favored for evaluating therapeutic efficacy because of its reliability in consistent tumor growth within a practical time frame of 4–5 weeks.

Other researchers have developed syngeneic ovarian cancer cell lines with defined genetic alterations relevant to high grade serous ovarian cancer, including p53, Brca1, myc, and Akt. The generation and thorough characterization of these cell lines, including BR-luc, SO, and SO1, has not been reported in a stand-alone publication, however, their use in FVB and C57BL/6 syngeneic models has contributed to key advances in the treatment of ovarian cancer, including targeting homologous recombination deficiency^7,8^, understanding microenvironment-driven immune suppression^9^, developing cancer vaccines^10^, and evaluating responses to immunotherapies^11,12^. Like the ID8 cell line, these genetically engineered cell lines reliably reproduce advanced ovarian cancer and develop ascites within a short time frame (10-40 days, depending on their genetic alterations). However, unlike the original ID8 cells and genetically modified ID8 cells, some of these genetically engineered cell lines have a high tumor mutation burden (TMB) and form extensive tertiary lymphoid structures^11,12^. These features enable detailed studies of immune escape mechanisms and provide a robust platform for assessing the efficacy of immunotherapies^11,12^.

As research shifted focus from the ovarian surface epithelium to the fallopian tube mucosa as the primary origin of most human ovarian cancers^13^, a new generation of syngeneic cancer cell line was developed by transforming murine fallopian tube epithelial cells with genetic alterations relevant to human tubo-ovarian cancer, including the loss of Brca1, Pten, Nf1, and Trp53 and overexpression of Trp53^R172H^, Ccne1, Akt2, Smarca4, and Kras^G12D14^. A major advantage of these cell lines lies in their alignment with the current understanding of tubo-ovarian cancer origins and their design as a panel of cell lines with progressively increasing genetic complexity. This structure allowed for a detailed analysis of how specific genetic alterations contribute to tumor growth and therapeutic responses, all thoroughly described in a single publication^14^. Potential disadvantages include variable tumor latency and the loss of epithelial morphology in some cell lines, which may limit their applicability in certain experimental contexts.

Although the existing syngeneic ovarian cancer cell lines were originally described as derived from either the mouse ovary or the fallopian tube, practical considerations raise questions about their true origin. Accurately isolating specific cell types from these tissues poses significant technical challenges, given the proximity of various epithelial and stromal cell populations and the absence of robust, definitive markers to precisely identify fallopian tube and ovarian surface epithelium during the cell isolation process. This makes it plausible that the cell lines were derived from transformed precursors to Pax8-positive epithelial cells (i.e., progenitor cells) rather than fully differentiated epithelial cells of the ovary or fallopian tube. Such precursor cells are more likely to undergo malignant transformation due to their higher plasticity and proliferative renewal potential.

With the plethora of syngeneic ovarian cancer cell lines to choose from, is there a need for additional cell lines? The effectiveness of currently available therapies for treating ovarian carcinomas remains limited, largely due to the heterogeneous nature of these tumors. Ovarian carcinomas encompass a variety of distinct mutant genotypes, each of which exerts diverse and often poorly understood influences on tumor phenotypes. These influences extend to critical factors such as the tumor’s microenvironment and its response to treatment. This complexity poses significant challenges, particularly in developing and evaluating new immunotherapies. Our limited understanding of the mechanisms by which cancer genotypes shape both immunophenotypes and therapeutic responses further complicates efforts to improve outcomes in this disease. Using multiple cell lines allows researchers to capture and study this diversity, leading to more accurate and generalizable findings. Herein, we describe the generation of a panel of syngeneic mouse ovarian epithelial cancer cell lines with diverse defined genetic alterations.

## Materials and methods

### Mouse strains

C57BL/6 and BALB/c nude mice were obtained from the Charles River Laboratories. B6.129x2-Trptm1 Tyj/J p53+/- heterozygote mice were obtained from the Jackson Laboratory and were bred and genotyped to select C57BL/6 p53-/- female offspring. All procedures in mice were performed in accordance with the NIH Guide for the Care and Use of Laboratory Animals and approved by the Cedars-Sinai Medical Center Institutional Animal Care and Use Committee (IACUC). The study is reported in accordance with ARRIVE guidelines.

### Isolation of mouse ovarian surface epithelial cells

Ovaries were removed from newborn B6.129x2-Trptm1 Tyj/J p53-/- female pups under aseptic conditions. Newborn mice were selected as the source of primary cells based on two main considerations. First, cells derived from newborn mice are generally more amenable to in vitro culture and expansion compared to those from adults. Second, newborn mouse ovaries have a higher epithelial-to-stromal cell ratio, increasing the likelihood of isolating and expanding ovarian surface epithelial cells with diminished interference from blood cells and fibroblasts. Ovarian surface epithelial cells were isolated as previously described^15^. Briefly, the ovarian bursa was cut and removed to expose the ovarian surface. Ovaries were transferred into a tissue culture dish and washed with PBS three times. Ovaries were treated with 0.025% trypsin for 60 minutes at 37 °C to detach the surface epithelial cells. Ovaries were removed from the dish with cells and growth medium (DMEM, containing 4.5 g/L glucose, L-glutamine, sodium pyruvate, 10% FBS, 1% penicillin/streptomycin antibiotics, 10 ng/ml EGF, 500 ng/ml hydrocortisone, and 10 µg/ml insulin) was added to dilute the trypsin. Cells were cultured in a humidified incubator at 37 °C in 5% CO_2_.

### Plasmids

pBabe-Puro (#1764), pWzl-Hygro (#18750), pWzl-Hygro-H-Ras V12 (#18749), pWzl-Blast (#12269), pWzl-Blast-Myc (#10674), pLenti-CMV-puro-Luc (w168-1) and pRcCMV-CCNE1 (#8963)^16^ were obtained from Addgene (Cambridge, MA, USA). The Khandan Keyomarsi laboratory (MD Anderson Cancer Center) kindly provided constructs for full-length (FL) cyclin E (pcDNA 3.1 cyclin E)^17,18^. CCNE1 cDNA was cloned into the pBABE-puro mammalian expression vector at the EcoRI site in the multiple cloning site to generate pBABE-CCNE1 and verified by sequencing. Pax2 packaging plasmid (#35002), and VSVG envelope plasmid were obtained from Addgene.

### Antibodies

For immunohistochemistry, the following antibodies were used: CA125 rabbit polyclonal (Abbiotec, San Diego, CA, USA), 1 mg/ml, 1:200 dilution; CK8 rabbit polyclonal (Bioss, Woburn, MA, USA), 1 µg/µL, 1:100 dilution; CK7 mouse monoclonal (OriGene, Rockville, MD, USA), 1ug/uL 1:100 dilution; C-Myc rabbit polyclonal (Santa Cruz Biotechnology, Dallas, TX, USA), 200 µg/ml, 1:200 dilution; E-cadherin, mouse monoclonal (BD Transduction Laboratories), 250 µg/ml, 1:1000 dilution; Estrogen receptor α, MC-20 rabbit polyclonal (Santa Cruz Biotechnology), 200 µg/ml, 1:200 dilution; H-Ras, C-20 rabbit polyclonal (Santa Cruz Biotechnology), 200 µg/ml, 1:200 dilution; CyclinE mouse monoclonal (Vector Laboratories, Burlingame, CA USA), 1:100 dilution; Pan-cytokeratin, C11 mouse monoclonal (Santa Cruz Biotechnology), 200 µg/ml, 1:200 dilution; PAX8 rabbit polyclonal (Epitomics, Burlingame, CA, USA ), 100 µg, 1:200 dilution; WT1 rabbit monoclonal (LSBio, Seattle, WA, USA ), 100 µL, 1:100 dilution; Ki67 mouse monoclonal (Abcam), 1:200 dilution; F4/80 rat anti-mouse (Caltag Medsystems, Ltd), 1:100 dilution; CD68 rat anti-mouse (Bio-Rad), 1:100 dilution; CD3 rat anti-mouse (BD Biosciences); 1:200; CD8 rat anti-mouse (Bio-Rad), 1:200. For Western blot analysis, anti-CyclinE-HE12 and HRas antibodies (Santa Cruz Biotechnology, Dallas, TX) were used at 1:500 dilution and the anti-cMyc antibody (Cell Signaling Technology, Danvers, MA) was used at 1:1000 dilution. Mouse Actin Antibody (Sigma) and rabbit actin antibody (Abcam) were used at 1:5000 dilution. IRDye-labeled secondary antibodies (LI-COR® Biosciences, Lincoln, NE, USA) were diluted 1:10,000.

### Retroviral transduction of TP53^-/-^ mouse ovarian cells

LinX-A cells were plated in 6-well plates at 2.5 x 10^5^ cells/well in DMEM containing 10% FBS. Retroviral vectors containing pertinent plasmids were transfected into 70% confluent LinX-A cells with Lipofectamine 2000 (Invitrogen, Grand Island, NY, USA). After 48 hours, viral supernatant was centrifuged at 1000 rpm and filtered through a 0.45 µm filter. TP53^-/-^ ovarian cells were plated in 6-well plates at 2.5 x 10^5^ cells/well in supplemented DMEM medium and grown for 2hours before viral transduction. Viral supernatant containing retroviruses of interest was added to TP53^-/-^ ovarian cells with growth medium containing 8 µg/ml of polybrene. Plates were centrifuged at 1700 rpm for 1 hour at room temperature and cells were grown in a humidified incubator at 37 °C and 5% CO_2_. The medium was replaced after 24 hours. Selection for successfully transduced cells was initiated after 48 hours using appropriate antibiotics (5 µg/ml puromycin, 5 µg/ml blasticidin, and 200 µg/ml hygromycin).

### Lentiviral transduction of MYC-HRAS-Luc mouse ovarian cells

293Tx packaging cells were plated in a 10 cm dish in DMEM, 10% FBS, and 1% penicillin/streptomycin medium. Upon reaching 70% confluency, cells were transfected with 3 lentiviral plasmids using standard lentiviral transduction protocols. Briefly, a mixture of 9 µg of packaging plasmid Pax2, envelope plasmid VSVG, and pLenti-puro-Luc plasmid in OPTI-MEM medium was mixed with Lipofectamine (27 µl) in OPTI-MEM and transferred into the packaging cells in growth medium without antibiotics. The cells were grown for 18 hours after transfection (37 °C, 5% CO_2_) when the medium with transfection reagents was replaced with 10 ml DMEM (15% FBS, 1% pen/strep) for viral production. After 24 hours, the medium was harvested, filtered through a 0.45 µm filter, and added to the MYC-HRAS cells with 8 µg/ml polybrene. The cells were grown in a humidified incubator at 37 °C and 5% CO_2_. After 24 hours, the medium was replaced with fresh medium and the selection of the Luciferase-expressing cells was initiated after 48 hours of cell growth by adding 5 µg/ml of puromycin to the transduced cells.

### Soft agar assay

6-well plates were coated with a base layer of 0.5% low-melting temperature agarose (SeaPlaque ® Agarose, Lonza Rockland, ME, USA) in DMEM media containing 10% FBS and 1% pen/strep antibiotics. Transduced mouse ovarian cells were resuspended in DMEM/FBS/antibiotics and 0.4% agarose. 30,000 cells per well in 3 ml were added to the base layer and incubated at 37 °C, 5% CO_2_. Colonies were counted after 14 days using a dissection stereomicroscope 24 hours after the addition of 0.2 ml of 1 mg/ml iodonitrotetrazolium chloride staining solution (Sigma-Aldrich, St. Louis, MO, USA).

### In vivo tumorigenicity assay

2x10^6^ of transduced mouse ovarian cells in 500 µl of PBS were injected into the peritoneal cavity of 6- to 8-week-old nude or C57BL/6 mice. Mice were monitored for tumor growth and euthanized when abdominal bloating was observed. Intraperitoneal tumors were harvested and collected for (1) growth in cell culture, (2) freezing in liquid nitrogen and storage at − 80 °C, and (3) fixation with formalin and paraffin embedding.

### Immunohistochemistry

Intraperitoneal tumors from each mouse were fixed in 4% formaldehyde for at least 48 hours at 4 °C, then transferred into 70% ethanol and submitted to the Department of Pathology and Laboratory Medicine at Cedars-Sinai Medical Center for paraffin-embedding, sectioning, and H&E staining. Immunohistochemistry on unstained slides was performed using the VECTASTAIN Elite ABC kits (Vector Laboratories, Burlingame, CA, USA), following the manufacturer’s instructions as previously described^19^. Briefly, sections were deparaffinized in a series of graded xylene/ethanol solutions and hydrated before antigen retrieval in citrate-based antigen unmasking solution (pH6) from Vector Laboratories for 15 minutes. Endogenous peroxidase was inactivated with 0.3% hydrogen peroxide in methanol. Slides were blocked with normal goat serum for rabbit primary antibodies or with MOM (mouse-on-mouse) IgG blocking reagent for mouse primary antibodies. Primary rabbit antibodies were diluted in 1% BSA/1% Tween 20/PBS solution. Primary mouse antibodies were diluted in MOM diluent solution. Slides were incubated with the primary antibody for 1 hour. Biotinylated secondary antibody was applied after treating the slides with the ABC reagent. Slides were stained with ImmPACT DAB solution (Vector Laboratories) according to the manufacturer’s instructions. The reaction was stopped with PBS washes and slides were counterstained with hematoxylin, dehydrated, and mounted.

### Western blot analysis

Protein content in whole cell lysates was determined by the BCA method (Thermo Scientific, Rockford, IL, USA). The proteins were separated on a 4–15% Tris/HCl polyacrylamide mini PROTEAN® TGX Precast Gel (Bio-Rad, Hercules, CA, USA) and transferred to a PVDF membrane using the Trans-Blot-Turbo Transfer System (Bio-Rad) for 10 minutes. The membrane was blocked for 1 hour using the Odyssey® blocking buffer. The membranes were incubated with primary antibodies in the blocking buffer with 0.1% Tween-20 overnight at 4^ °^C. After washing in PBST buffer, the membrane was probed with Infrared IRDye-labeled secondary antibody (LI-COR ® Biosciences, Lincoln, NE, USA) in blocking buffer with 0.1% Tween-20 for 45 minutes. The protein bands on the membrane were visualized using the Odyssey® Infrared Imager (LI-COR ® Biosciences).

### Animal imaging

Luciferase-expressing MYC-HRAS cells were injected i.p. (1x10^6^) into C57BL/6 mice. Five days after the injection of cells, tumor growth was monitored using the Xenogen intravital imaging system as previously described^20^.

### Statistical analyses

The statistical analyses were performed using GraphPad Prism (version 6.0; GraphPad Software).

## Results

### Development of genetically defined epithelial ovarian cancer cell lines

**Fig. 1:**
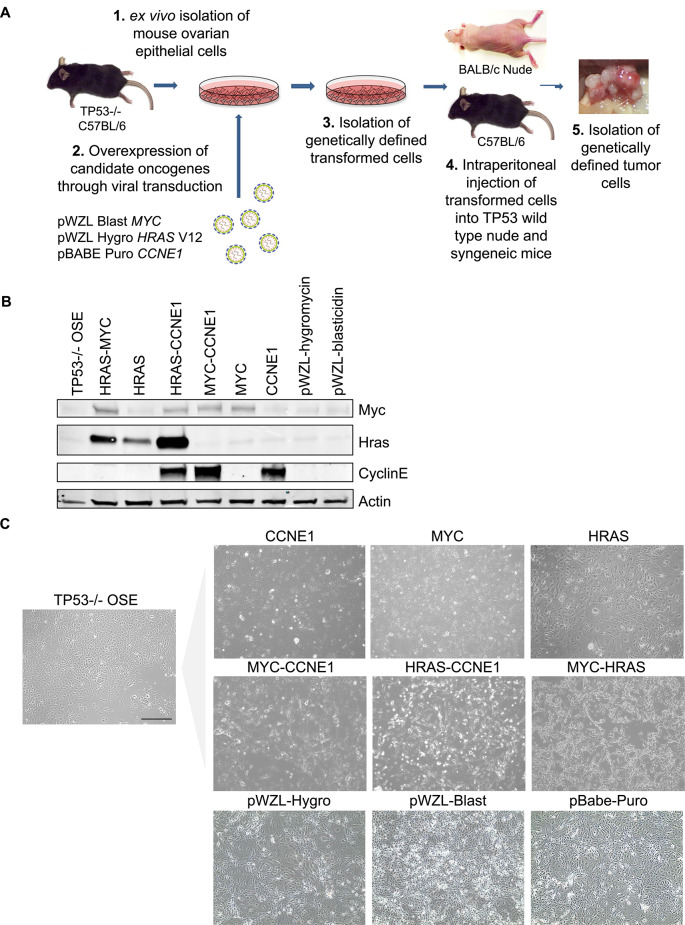
Syngeneic mouse ovarian cancer cell lines. (**A**) Generation of syngeneic mouse ovarian cancer cell lines with defined genetic alterations. (**B**) Western blot analysis of protein expression in genetically defined cell lines. (**C**) Morphology in cell culture; scale bar: 100 µm.

TP53^-/-^ cells isolated from the ovaries of TP53^-/-^ C57BL/6 mice were transduced with viral vectors containing oncogenes HRAS^V12^, MYC, and CCNE1, individually or in combination (Fig. 1A). The expression of transduced proteins was confirmed by Western blot analysis (Fig. 1B and Supplementary Fig. 1). The transduced TP53^-/-^ cells lost the typical cobblestone morphology of mouse ovarian cells in 2D culture (Fig. 1C). MYC and HRAS transduced TP53^-/-^ cells had drastically different phenotypes; MYC cells grew as a tight monolayer of epithelial cells while HRAS cells comprised large polynucleated cells that resembled senescent cells or polyploid giant cancer cells (PGCs)^21^ (Fig. 1C). The morphology of MYC-HRAS cells was different from cells transduced with individual oncogenes; MYC-HRAS cells exhibited spindle cell morphology or grew as clusters of rounded cells loosely attached to the substrate (Fig. 1C). All cell lines except CCNE1 transduced cells formed anchorage-independent colonies in soft agar after 3 weeks (Fig. 1C).

### Tumorigenicity and tumor latency in nude mice

**Fig. 2:**
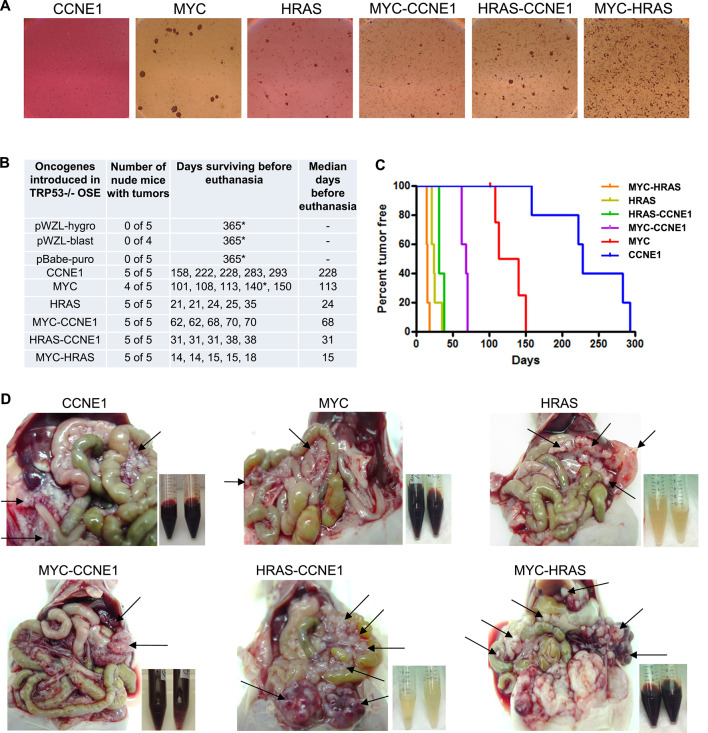
Cell line growth in soft agar and tumor formation in nude mice. (**A**) Anchorage-independent formation of foci in soft agar (day 14 after seeding). (**B**) Latency of tumor development in nude mice. (**C**) KM plot in nude mice. (**D**) Tumor growth patterns in nude mice.

All cell lines except CCNE1 transduced cells formed anchorage-independent colonies in soft agar after 3 weeks (Fig. 2A). The tumorigenic potential of transduced TP53-/- cells was evaluated through intraperitoneal (i.p.) injection into athymic nude mice. Tumor latency in nude mice is depicted by Kaplan–Meier survival curves (Fig. 2B, C). In all cases, tumors disseminated throughout the peritoneal cavity, localizing to the diaphragm, omentum, bowel mesentery, and pelvis (Fig. 2D), recapitulating the dissemination pattern of human ovarian cancer. Each oncogene in the TP53-/- context induced tumor formation with distinct latency and phenotypic characteristics. CCNE1-transduced cells formed tumors with hemorrhagic ascites in 100% of mice at a median latency of 228 days (range: 158–293 days), suggesting the requirement of additional genetic alterations for tumorigenesis. MYC-transduced cells induced microscopic tumors and hemoperitoneum (without ascites) in 80% of mice at a median latency of 113 days (range: 102–150 days). HRAS-transduced cells caused macroscopic intraperitoneal tumors with clear ascites in 100% of mice at a median latency of 24 days (range: 21–35 days). Co-expression of oncogenes resulted in accelerated tumorigenesis. MYC-CCNE1-transduced cells formed tumors in 100% of mice at a median latency of 68 days (range: 62–70 days), suggesting synergistic cooperation between these oncogenes. Similarly, HRAS-CCNE1 cells caused tumors with clear ascites in 100% of mice at a median latency of 31 days. MYC-HRAS-transduced cells exhibited the most rapid tumor formation, producing macroscopic tumors with hemorrhagic ascites in 100% of mice at a median latency of 15 days (range: 14–18 days). This phenotype represented an acceleration compared to single oncogenes, with a shift from clear ascites (seen in HRAS-transduced tumors) to hemorrhagic ascites and from microscopic tumors (seen in MYC-transduced tumors) to macroscopic lesions. In contrast, no tumor formation was observed within 365 days in nude mice injected with TP53-/- cells transduced with empty vectors (pBABE-puro, pWZL-hygro, or pWZL-blast) and passaged 14–34 times in culture. Collectively, these findings demonstrate that while a single oncogenic driver (CCNE1, MYC, or HRAS) is sufficient to transform TP53-/- cells, the co-expression of two oncogenes enhances tumorigenicity through distinct, cooperative pathways, impacting tumor latency and phenotype.

### Tumors recapitulate immunohistological characteristics of human epithelial ovarian cancer

**Fig. 3:**
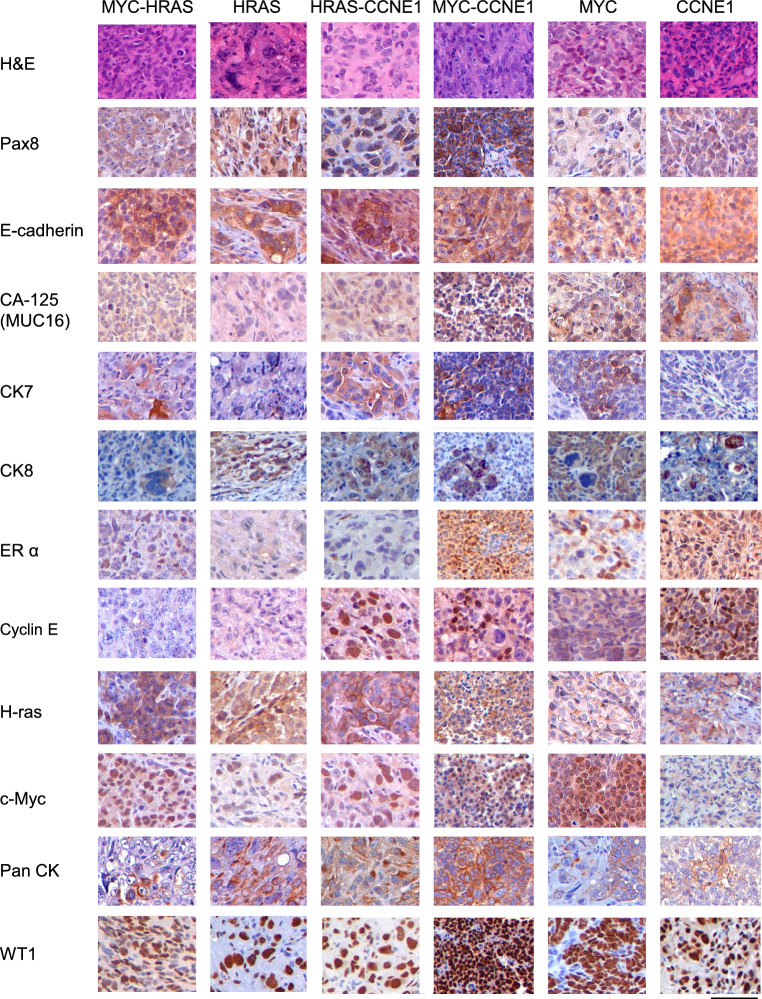
Immunohistochemical markers. Immunohistochemical analysis of cancer markers; scale bar: 50 µm.

Histological examination of tumors derived from transduced TP53-/- cells revealed features consistent with undifferentiated or high-grade carcinoma (Fig. 3). Tumors predominantly comprised solid sheets and nodules of pleomorphic epithelioid cells with pronounced nuclear atypia. Immunohistochemical analysis confirmed that tumors expressed Cyclin E, H-Ras, or c-Myc, correlating with the oncogene used for transduction (Fig. 3). PAX8 staining was predominantly cytoplasmic rather than nuclear, which is atypical for a transcription factor. This pattern of expression could have emerged due to oncogenic stress, especially as tumors adopt mesenchymal, stem-like, or poorly differentiated phenotypes. While PAX8 is a widely used marker of Müllerian lineage and fallopian tube-derived HGSOC, it has been shown to be weak or absent in tumors derived from ovarian surface epithelium (OSE), which does not naturally express PAX8^22^. Importantly, despite this atypical localization of PAX8, the tumors expressed pan-cytokeratin (a marker of epithelial differentiation) and WT1 (a marker of serous ovarian carcinoma).

### Syngeneic ovarian cancer models

**Fig. 4:**
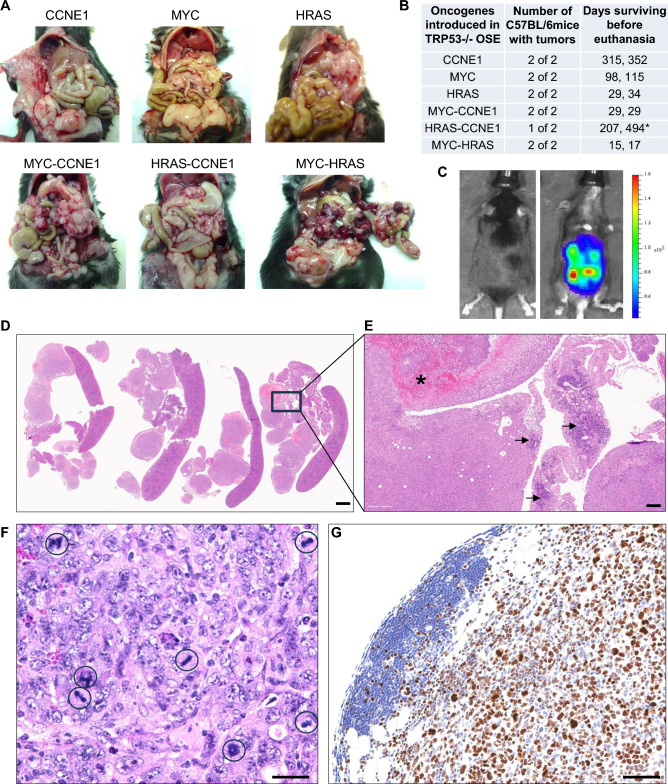
Tumor growth in immunocompetent mice. (**A**) Tumor growth patterns in immunocompetent C57BL/6 mice. (**B**) Latency of tumor development. (**C**) IVIS imaging of GFP-Luc transduced MYC-HRAS cells. (**D**) H&E section, MYC-HRAS cells intraperitoneal tumor nodules in the omentum and spleen; scale bar: 2 mm. (**E**) H&E section, formation of tertiary lymphoid structures (arrows) and necrosis (asterisk) in the MYC-HRAS model; scale bar: 500 µm. (**F**) H&E section, high magnification of tumor cells in the MYC-HRAS model reveals nuclear atypia, pleomorphism, mitotic figures, and pyknotic nuclei; scale bar: 50 µm. **(G)** Ki67 immunostaining signal in the MYC-HRAS model; scale bar: 100 µm.

**Fig. 5:**
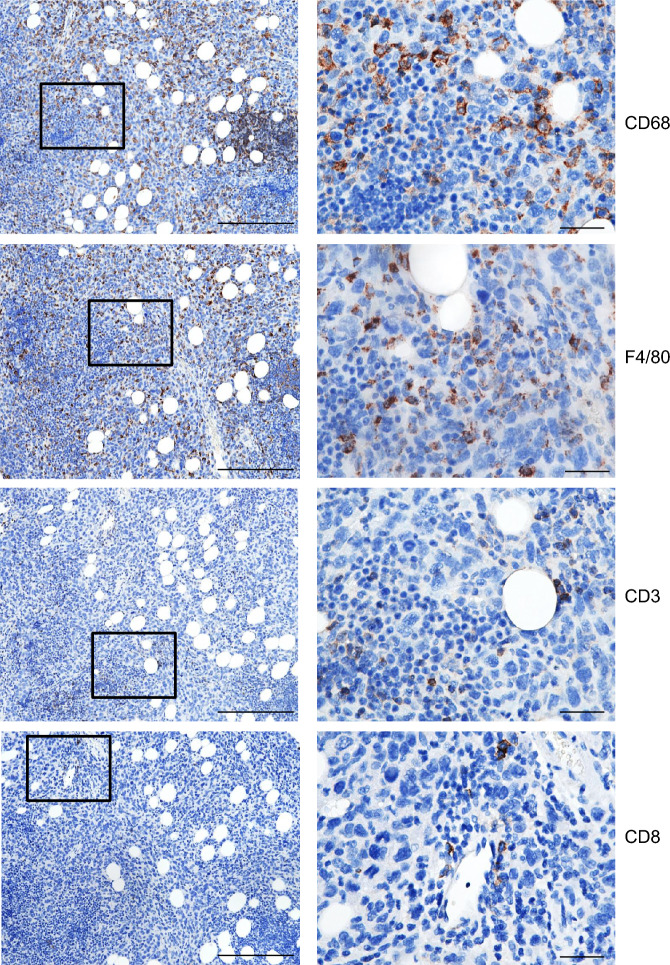
Immunohistochemical characterization of immune cell infiltration in tumors. Immunohistochemical analysis of intraperitoneal MYC-HRAS cancers with markers of macrophages/monocytes (CD68 and F4/80) and T cells (CD3 and CD8); scale bars: 250 µm for panels on the left and 50 µm for panels on the right.

Female C57BL/6 mice i.p. injected with TP53-/- cells transduced with oncogenes developed tumors exhibiting a similar phenotype and latency to those in nude mice, with the exception of HRAS-CCNE1 cells, which displayed poor growth (Fig. 4A, B). MYC-HRAS cell lines were additionally transduced with luciferase for intravital imaging (Fig. 4C). Notably, MYC-HRAS tumors demonstrated a short latency period (2–3 weeks) and histologic analysis revealed prominent hypoxic/necrotic regions and abundant immune cell infiltrates (Fig. 4D, E). These tumors were characterized by marked cellular and nuclear atypia, as well as frequent mitotic figures (Fig. 4F). Ki67 immunostaining further confirmed the high proliferative activity of the tumor cells (Fig. 4G). Immunohistochemical analysis of macrophage/monocyte markers CD68 and F4/80 and T cell markers CD3 and CD8 reveals abundant tumor infiltration by macrophages/monocytes and sporadic infiltration by T cells (Fig. 5).

## Discussion

Syngeneic mouse ovarian cancer cell lines are critical tools for advancing our understanding of ovarian cancer biology and treatment. By providing a platform to study tumor-immune interactions and evaluate novel therapies, these models bridge the gap between preclinical research and clinical application. Continued refinement and innovation in syngeneic models are essential for improving outcomes in ovarian cancer patients. Herein, we report on the development and characterization of robust genetically defined models of epithelial ovarian cancer. We propose this as a versatile model system to address the interplay between different patterns of oncogene activation on subsequent ovarian cancer phenotype and response to targeted therapies. Variations in tumor onset latency enable the selection of the most suitable cell line for specific preclinical trials.

We have demonstrated that overexpression of a single candidate oncogene, or a combination of oncogenes, such as activated HRAS, MYC or CCNE1 can transform TP53-/- cells into serous or undifferentiated epithelial ovarian cancers. The latency period to intraperitoneal tumor formation and tumor phenotype differed based on the activated oncogene or combination of oncogenes introduced. Tumors expressed high levels of the introduced oncogene protein products by immunohistochemistry and TP53-/- cells transduced with empty lentiviral vectors did not cause transformation, suggesting that tumor development was driven by oncogenic drivers. The prolonged latency observed in the CCNE1-HRAS double mutant group compared to the HRAS single mutant was unexpected. One possible explanation is that overexpression of CCNE1 in the context of oncogenic HRAS may trigger oncogene-induced senescence (OIS) or another intrinsic tumor-suppressive mechanism, such as replication stress or DNA damage response activation, which can delay or prevent tumor formation. This phenomenon has been observed in other models where strong proliferative signals paradoxically suppress tumorigenesis^23^.

We have previously reported that transduction of ovarian TP53-/- cells with single oncogenes, such as MYC, KRAS^G12D^, and myristoylated AKT, is insufficient to produce tumors in nude and immunocompetent mice within 180 days^24^. However, in this report, we show that MYC-transduced TP53-/- cells form tumors with an average onset of 113 days. The two studies used different cell-transduction techniques (avian RCAS viruses vs. lentiviruses). One possible explanation for the discrepancy in the ability of myc overexpression to transform TP53-/- cells is that cells from TP53-/- TVA-transgenic mice in the previous report were directly i.p. injected into mice after a five-day incubation with Replication-Competent ALV (Avian Leukosis Virus) with a Splice acceptor (RCAS) viruses, without further selection and passaging in culture, while the lentiviral transduction in this report required antibiotic selection and expansion of selected cells. It is possible that the freshly transformed cells were less efficient at engrafting in the new environment than the cells that had time to recover during selection and in vitro passaging. It is also likely that secondary genetic alterations occurred during antibiotic selection and in vitro or in vivo growth, especially in cell lines that exhibited long tumor latency. However, the development of tumors in 100% of mice tested suggests that the introduced genetic alterations in the setting of TP53 deficiency create a foundation for these secondary genetic events.

## Conclusions

All of the genetically defined cell lines discussed here will be valuable for studying individual genes in tumor progression and response to targeted therapies. The MYC-HRAS model will be particularly crucial for investigating genomic instability and immunotherapy responses. Notably, this model is highly aggressive and genomically unstable, with tumors detectable via luciferase imaging just five days after intraperitoneal injection of 1x10^6 cells into C57BL/6 mice. Compared to ID8 cells, MYC-HRAS cells form tumors with shorter latency and 100% penetrance (unpublished observations). A key distinguishing feature of MYC-HRAS cells, unlike ID8, is the formation of tertiary lymphoid structures. Given the growing interest in the role of tertiary lymphoid structures in ovarian cancer progression and immune response, we believe this model will be invaluable for in vivo studies of how immunotherapies impact tertiary lymphoid structures formation and organization. Additionally, a valuable resource developed in this study are the TP53-/- nontumorigenic primary ovarian epithelial cells, which can be cultured and efficiently transduced with lentiviral vectors carrying specific genetic alterations. These cells offer an excellent platform for generating further models that mimic various genetic events in human ovarian cancer.

## Supplementary Information

Supplementary Material 1.

## Data Availability

The representative data from this study are included in the article; further inquiries can be directed to the corresponding author.
