# Supplementary material for: Immunocompetent C57BL/6 syngeneic mouse ovarian cancer models with defined genetic alterations
Source: Sci Rep. 2025 Oct 7;15:34969. doi: 10.1038/s41598-025-18960-5 (PMC12504517; doi:10.1038/s41598-025-18960-5)
Supplement: Supplementary file 1 — Supplementary Material 1. [file 41598_2025_18960_MOESM1_ESM.pdf]

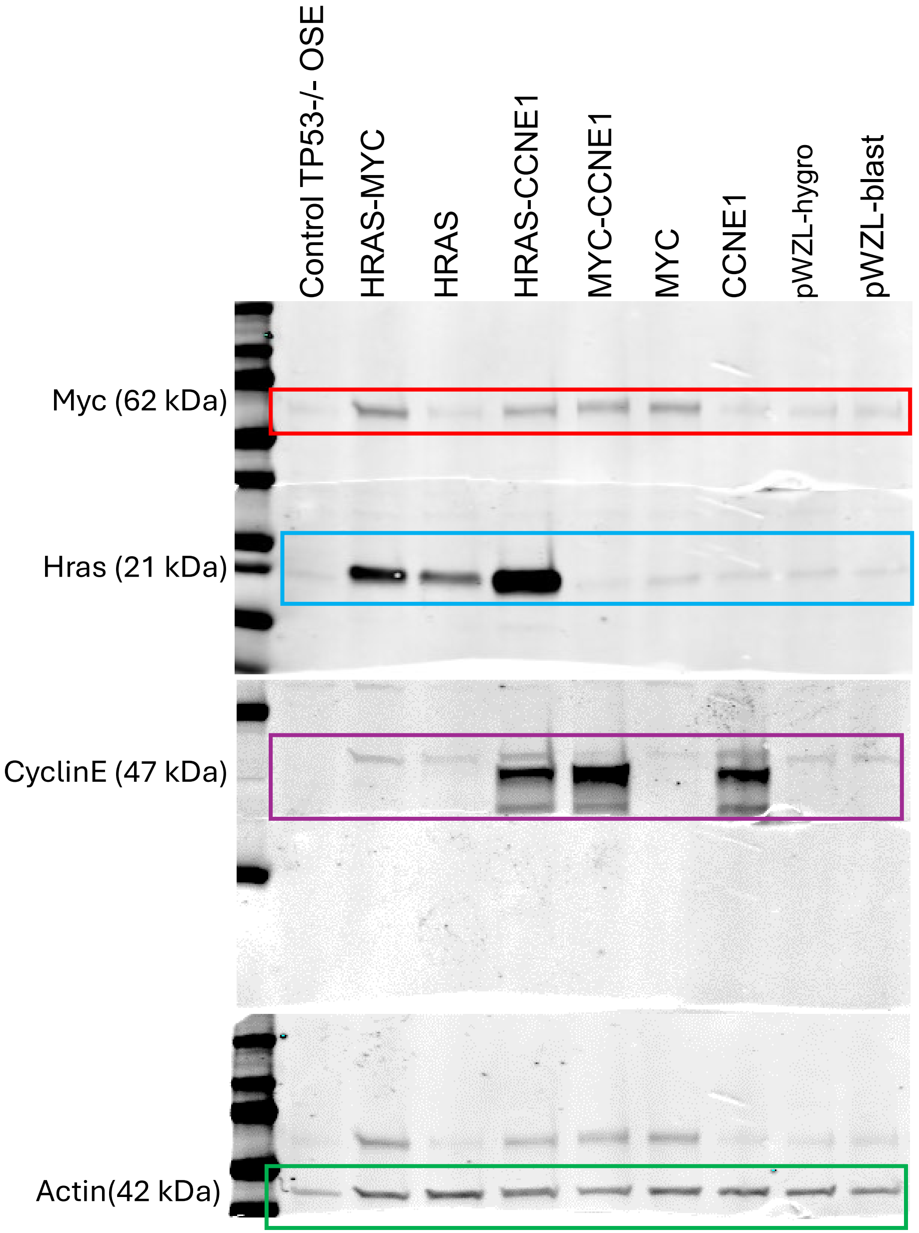

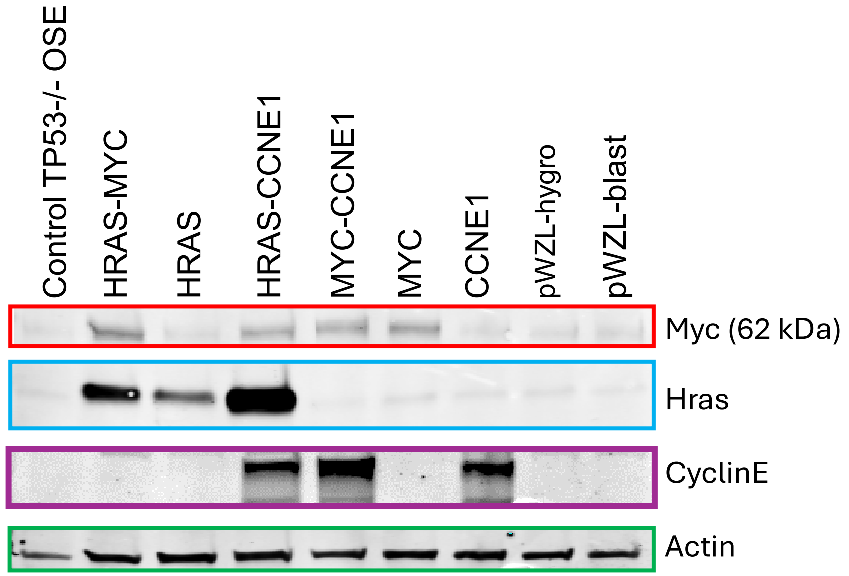

From Fig. 1B.

Suppl. Fig. 1. Uncropped Western blots. Color coding is used to link the cropped blots in Fig. 1B to their corresponding uncropped original membrane blots.
